# Sexual Dimorphism of Cranial Morphological Traits in an Italian Sample: A Population-Specific Logistic Regression Model for Predicting Sex

**DOI:** 10.3390/biology11081202

**Published:** 2022-08-10

**Authors:** Annalisa Cappella, Barbara Bertoglio, Matteo Di Maso, Debora Mazzarelli, Luciana Affatato, Alessandra Stacchiotti, Chiarella Sforza, Cristina Cattaneo

**Affiliations:** 1 Dipartimento di Scienze Biomediche per la Salute, Università degli Studi di Milano, Via Mangiagalli 31, 20133 Milan, Italy; 2 U.O. Laboratorio di Morfologia Umana Applicata, IRCCS Policlinico San Donato, 20097 San Donato Milanese, Italy; 3 LABANOF (Laboratorio di Antropologia e Odontologia Forense), Sezione di Medicina Legale, Dipartimento di Scienze Biomediche per la Salute, Università degli Studi di Milano, Via Mangiagalli 37, 20133 Milan, Italy; 4 Department of Clinical Sciences and Community Health, Branch of Medical Statistics, Biometry and Epidemiology “G.A. Maccacaro”, Università degli Studi di Milano, Via Vanzetti 5, 20133 Milan, Italy; 5 LAFAS (Laboratorio di Anatomia Funzionale dell’Apparato Stomatognatico), Dipartimento di Scienze Biomediche per la Salute, Università degli Studi di Milano, Via Mangiagalli 31, 20133 Milan, Italy

**Keywords:** sexual dimorphism, cranial morphological traits, Collezione Antropologica Labanof (CAL), population-specific equations, logistic regression models, sex estimation, accuracy

## Abstract

**Simple Summary:**

Despite the fact that sex estimation methods from crania are very popular in forensic anthropology, few validation studies have verified their accuracy and reliability in different populations. Different from craniometrics, for which validation studies have remarkably increased lately, the methods based on cranial morphology still need to be thoroughly investigated, even if a large consensus exists on the effects of population variability on sexual cranial dimorphism. When dealing with forensic contexts, appropriately-validated methods should be applied for building accurate biological profiles. Since the possible sexual dimorphism variation of cranial morphological traits needs to be evaluated properly in various populations, in this study, we analyzed the accuracy of existing regression models for predicting sex from cranial morphological traits in an Italian contemporary/modern population. In addition, we propose new logistic regression models that are more accurate and specific for our sample. The results also update the reference standards for populations of this geographical area and provide an additional important warning on sexual dimorphism to anthropologists working in forensic contexts.

**Abstract:**

Although not without subjectivity, the cranial trait scoring method is an easy visual method routinely used by forensic anthropologists in sex estimation. The revision presented by Walker in 2008 has introduced predictive models with good accuracies in the original populations. However, such models may lead to unsatisfactory performances when applied to populations that are different from the original. Therefore, this study aimed to test the sex predictive equations reported by Walker on a contemporary Italian population (177 individuals) in order to evaluate the reliability of the method and to identify potential sexual dimorphic differences between American and Italian individuals. In order to provide new reference data to be used by forensic experts dealing with human remains of modern/contemporary individuals from this geographical area, we designed logistic regression models specific to our population, whose accuracy was evaluated on a validation sample from the same population. In particular, we fitted logistic regression models for all possible combinations of the five cranial morphological traits (i.e., nuchal crest, mastoid process, orbital margin, glabella, and mental eminence). This approach provided a comprehensive set of population-specific equations that can be used in forensic contexts where crania might be retrieved with severe taphonomic damages, thus limiting the application of the method only to a few morphological features. The results proved once again that the effects of secular changes and biogeographic ancestry on sexual dimorphism of cranial morphological traits are remarkable, as highlighted by the low accuracy (from 56% to 78%) of the six Walker’s equations when applied to our female sample. Among our fitted models, the one including the glabella and mastoid process was the most accurate since these features are more sexually dimorphic in our population. Finally, our models proved to have high predictive performances in both training and validation samples, with accuracy percentages up to 91.7% for Italian females, which represents a significant success in minimizing the potential misclassifications in real forensic scenarios.

## 1. Introduction

One of the critical aspects concerning the application of sex diagnostic methods to human remains is the inter-population variation in sexual dimorphism [1]. Indeed, the size and shape of some skeletal traits showing sexual dimorphism in one population may be much less dimorphic in a different geographical one or may exhibit secular changes in the same local population over time spans of a few decades [2,3,4,5]. In these perspectives, appropriate reference skeletal collections offer the chance to build specific ad hoc methods in which the problems of bias of sex estimation—in terms of spatial and temporal variation—may be avoided in part, thus along one to assess the sex accurately on human remains of individuals coming from the same, or similar, population. Hence, population-specific sexual dimorphic characteristics should be evaluated systematically in any documented reference skeletal population in order to create good standards applicable to real forensic casework [6,7]. The implementation of this approach is useful both for metric and morphological traits, with the intent to provide important informative data and supplementing methods to apply in cases characterized by few isolated or taphonomically modified elements in which the routine methods might not be applicable [8]. Therefore, it is important to develop population-specific methods for a variety of skeletal preservation scenarios. Alternatively, to provide, in any case, the most accurate data for any population [9].

Although the visual methods based on the morphological sex dimorphism of specific skeletal traits were among the first applied for assessing sex into the field of anthropology [7,10,11,12,13,14], their statistic quantitative approach is still less advanced compared to the analogous metric methods, which quickly became popular when developed with the use of discriminant functions as a statistical approach [15,16]. This is particularly due to the problem of subjectivity that characterizes such analyses: thus, the issue of observer repeatability is a problem not to underestimate [17] and is especially evident for the assessment of some specific skeletal traits, for instance, those of the cranium [18]. Nevertheless, in the past decades, the morphological variables of the cranium have restarted to gain significant attention in the literature [2,7,17,18,19,20,21] and are now combined with more complex statistical approaches that generate accurate discriminative models and formulae for specific populations. Walker [2] was one of the first authors to suggest the use of logistic regression for assessing the sex from categorical cranial morphologic traits in White and Black Americans and in an English sample. Since his study, some authors have started to assess the accuracy of Walker’s [2] equations in various groups [18,22,23], highlighting doubts about the reliability of Walker’s method due to the critical subjectivity intrinsically existing in morphological methods and to the inter-population variation. Some other methodological approaches consist of the use of machine learning techniques (i.e., decision analysis) in order to increase the accuracy of the predictive models [24]. Regardless of the model used to predict sex, the literature shows that not all morphological traits are performed equally, and methods should weigh every single trait accordingly. The different reliability of each cranial trait has been associated with the lack of representation in the variation extent by the scoring system. In other words, some highly variable features may be due, in part, to subjective scoring, which impacts reliability and accuracy. However, weighing the traits should not only be limited to a mere matter of a scoring system but, above all, should be extended to the existence of the sexual dimorphism variation of some traits among diverse populations, which is the real key point.

Kruger et al. [21] tested the reliability and accuracy of Walker’s formulae and the frequencies of the diverse scores for the cranial traits in a South African population (Black people and White people) with the intent to examine patterns of sexual dimorphism in this specific population: Walker’s equations resulted in low accuracies for South Africans, driving the authors to implement a logistic regression analysis to formulate their own modified equations. Similarly, Oikonomopoulou et al. [25] tested Walker’s equations in a modern Greek population. Again, the equations resulted in low accuracy for the Greek sample: thus, the authors developed and proposed their own modified equations and suggested extending this approach to other Balkan groups.

Given the inter-population variation, the application of the equations proposed by Walker may lead to inaccurate results on the remains of geographical groups that are different from the tested, thus providing low rates of correct sex diagnosis. Hence, an examination to verify this aspect is necessary for other diverse osteological collections. While this critical aspect has already been investigated in some populations [21,22,23,25], many worldwide groups have yet to be studied. Among these, Collezione Antropologica Labanof (CAL)—a documented skeletal collection recently assembled and represented mostly by historical, modern, and contemporary Italian individuals [26,27,28]—has been recently analyzed for the sexual dimorphism of cranial metric variables [29,30] but no investigation has been conducted on the cranial morphological traits.

Thus, the present study aims to: (i) test the repeatability of the assessment of morphological traits on crania of modern Italians from the CAL; (ii) test the accuracy of Walker’s equations on this population (by calculating the correct success rate); and (iii) create ad hoc discriminative equations for the Italian population through logistic regression analysis with the intent to identify which morphological traits provide the most accurate prediction for assessing the sex on the crania of individuals from this geographical area.

## 2. Materials and Methods

### 2.1. Study Sample

A sample of 177 crania was selected from known individuals of CAL [26], housed at the University of Milan. The CAL skeletal collection includes unclaimed contemporary cemeterial remains granted from the cemeteries of Milan to the university for educational and research purposes, in agreement with the National Police Mortuary Regulation (DPR 09.10.1990 No. 285, art. 43). The cemetery collection is composed mostly of Italian adult individuals, for which demographic data (sex, age, Italian origin, date of birth, and date of death) and death certificates or autopsy reports are available. More information on the demographic composition is detailed in [26]. The sample selected consists of crania from 84 females (47.5%) and 93 males (52.5%) with ages ranging from 18 to 96 years (mean ± standard deviation: 64 ± 20 years). The inclusion criteria were: (i) presence and good taphonomic state of preservation of the cranium; (ii) adult age (≥18 years); (iii) known demographic information (i.e., sex and age); (iv) good conservation of the five cranial morphological traits. Individuals with previous craniofacial trauma, cranial malformations, and pathological signs involving the cranium were excluded. For each selected cranium, the demographical data were checked in order to create a well-representative sample of a modern/contemporary Italian population: all of the individuals were Italians born in the 20th Century.

### 2.2. Assessment of Cranial Morphological Traits

**Figure 1:**
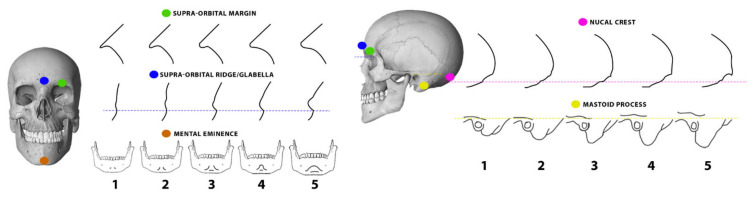
The five points-scale scoring system for the five cranial traits according to Walker’s classification [2].

The five points-scale scoring system described by Walker [2] and reported in Figure 1 was applied by two different analysts for scoring the morphology of all five cranial traits in each selected individual. The cranial morphological traits included the nuchal crest, mastoid process, orbital margin, glabella, and mental eminence. The lower the score, the minimally expressed and least pronounced the trait, with more gracile and robust features scored, respectively, as 1 (minimal expression) and 5 (maximal expression). Two analysts with different levels of experience (analyst 1: undergraduate level; analyst 2: post-doctoral level) independently applied the scoring system to the whole sample for each cranial morphological trait. Each cranium was scored independently, and its morphological traits were scored in the same order across the sample with no chance to re-examine the previously analyzed skulls. Agreements of scores between analysts were evaluated.

### 2.3. Statistical Analysis

The reproducibility (i.e., the interobserver agreement between the two analysts) of each cranial morphological trait score was assessed using the whole study sample, whereas the repeatability (i.e., the intraobserver agreement) was evaluated using a subsample of 50 crania. In particular, the assessment of cranial morphological trait scores for repeatability was conducted over a period from 1 to 3 weeks. The intra- and interobserver agreements were evaluated using the percentages of agreement with a tolerance of 0, and its degree was measured by weighted Cohen’s kappa [31], which is a variant of the classical Cohen’s kappa used to evaluate the agreement between two measurements expressed on an ordinal scale scoring system. Briefly, the weighted Cohen’s kappa takes the degree into account of disagreement between categories of an ordinal variable using a weighting scheme. In particular, the weighted Cohen’s kappa is calculated using a predefined table of weights that measures the degree of disagreement between the two evaluations; the higher the disagreement, the higher the weight. The interpretation of Cohen’s kappa statistics was carried out following Landis and Koch [32], who defined values <0 as no agreement, 0–0.20 as slight, 0.21–0.40 as fair, 0.41–0.60 as moderate, 0.61–0.80 as substantial, and 0.81–1 as almost-perfect agreement.

We reported absolute frequencies and proportions for age and each cranial morphological trait score according to sex. The differences in proportions according to sex were evaluated by means of a chi-squared test.

The linear relationship between each pair of cranial morphological trait scores was assessed by Spearman’s correlation coefficient.

The six discriminant equations proposed by Walker and developed on the American White/European sample [2] were applied to our whole sample with the intent to evaluate their accuracy on the Italian population. The accuracy was evaluated in terms of the percentage of correctly classified crania according to sex and the bias of the correct assessment between the two sexes (i.e., percentage of correctly classified female crania minus the percentage of correctly classified male crania).

Finally, in order to predict sex for the set of cranial morphological traits of the Italian population, we performed logistic regression models specific to this population. Logistic regression allows for an analysis of the association between a binary outcome (with two mutually exclusive levels) and one or more covariates, which may be either categorical or continuous [33]. In order to prevent overfitting and perform an accurate classification, we randomly split the whole study sample into a training sample accounting for 70% of the data (124 crania: 60 females and 64 males) and a validation one accounting for the remaining data (53 crania: 24 females and 29 males). We used the training sample to fit logistic regression models for all possible combinations of the five cranial morphological trait scores and the validation sample to provide an unbiased evaluation (i.e., the percentage of correctly classified skulls according to sex) of the fitted models. The cut point to predict sex was set to 0.5. Predicted probabilities greater than 0.5 suggest that the crania are more likely male, while predicted probabilities lower than 0.5 indicates that the crania are more likely female. All of the analyses were conducted in R, version 4.0.5 (R Core Team 2021).

## 3. Results

**Figure 2:**
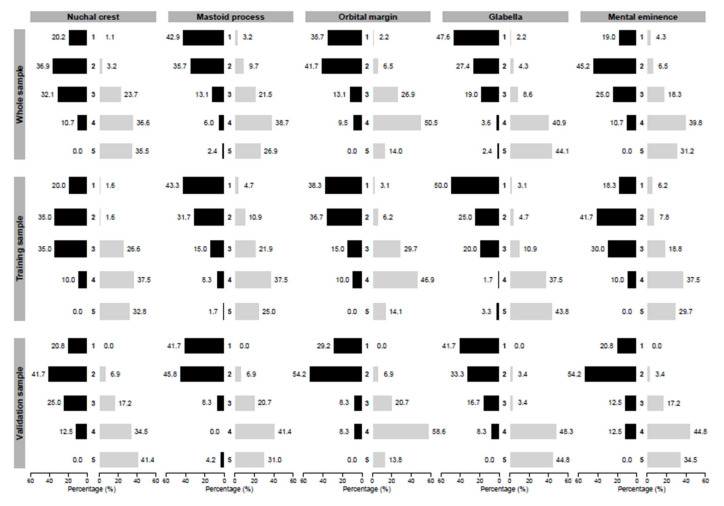
Distribution of cranial morphological trait scores (expressed in percentages) for females (grey bars) and males (black bars) in the whole, training, and validation samples.

Overall, the females were significantly older than the males (*p* < 0.01). The mean ± standard deviation of the age for females and males were 68.1 ± 20.1 and 60.3 ± 19.1 years, respectively. Figure 2 reports the bar plots depicting the distributions of each cranial morphological trait score according to sex separately for the whole, training, and validation samples. The distributions of all of the cranial morphological trait scores were significantly different (as reported by the *p*-values in Table 1) between males and females for the whole, training, and validation samples. In particular, male skulls received higher scores for all cranial morphological traits than female skulls.

Females showed significantly-positive linear relationships for all pairs of cranial morphological trait scores (*p* < 0.01; Table 2); males showed a similar pattern, except for the relationship between the nuchal crest and mental eminence that was not statistically significant (*p* = 0.99; Table 2). Similar relationships were observed in the training sample. Weaker relationships were observed in the validation sample, and some of these were not statistically significant.

Table 3 reports the inter- and intra-observer agreement of cranial morphological trait scores, where analyst 1 represents the undergraduate analyst and analyst 2 the post-doctoral one. The agreement between the two analysts (i.e., inter-observer agreement) ranged from 55.4% for the nuchal crest to 65.5% for the glabella when considering a tolerance of 0 degrees. The weighted Cohen’s kappa statistics showed a substantial/almost perfect agreement for all cranial morphological trait scores with values higher than 0.70. The two analysts showed similar intra-observer agreement for the nuchal crest (55.4% for analyst 1 and 59.6% for analyst 2), mastoid process (60.7% for analyst 1 and 68.1% for analyst 2), and mental eminence (67.9% for analyst 1 and 68.1 for analyst 2). The corresponding weighted Cohen’s kappa statistics were 0.70 (analyst 1) and 0.85 (analyst 2) for the nuchal crest, 0.88 (analyst 1) and 0.94 (analyst 2) for the mastoid process, and 0.81 (analyst 1) and 0.87 (analyst 2) for the mental eminence, indicating again a substantial/almost perfect agreement. Analyst 2 showed an almost perfect intra-observer agreement for the orbital margin (78.7%; weighted Cohen’s kappa = 0.90) and the glabella (83.0%; weighted Cohen’s kappa = 0.96), and overall, a higher intra-observer agreement than analyst 1.

The six Walker logistic regression equations applied to our sample correctly classified the sex with percentages between 74.6% and 85.9%, as reported in Table 4. However, correct classifications were much lower for females (56.0%–78.6%) than for males (90.3%–95.7%).

Table 5 reports the estimates of the logistic regression models for predicting sex according to all possible combinations of cranial morphological trait scores. In the models considering one cranial morphological trait at a time, all of the traits were significantly associated with sex. The model that included the mastoid process showed the best predicting performance with percentages of correctly classified skulls using data of the validation sample of 87.5% for females and 93.1% for males. Furthermore, the model including orbital margin showed a good predicting performance with 83.3% and 93.1% of correctly classified skulls for females and males, respectively. Among all fitted models, the best predicting performance was observed for models including nuchal crest and glabella and including mastoid process and mental eminence. Both models correctly classified 91.7% of the females and 93.1% of the males. Adding other cranial morphological traits to the previous models did not increase the prediction performance.

## 4. Discussion

The present study investigated morphological sexual dimorphism in the crania of adult humans from a geographically-homogeneous contemporary Italian sample. Sexual dimorphism in humans is the product of different growth and development patterns, which often follow geographic variations in environmental and genetic factors [34,35]. The cranium has regions which differ in ontogeny and function [36] and are thus expected to change and develop with diverse independence and degree of modularity [37]. As a result, differences in morphology and dimension exist between the crania of different individuals, the females and males within a single population, and between different populations [38,39].

In addition to geographical differences in sexual dimorphism, temporal differences likewise influence cranial morphology [5]: if one can assume that a population has differences in some skeletal traits when compared with another living in a geographically distant area from a genetic and phenotypical point of view, the same assumption can also be applicable for temporally-diverse populations. Indeed, individuals from a past population (that lived, for instance, 200 years ago) will be genetically and morphologically different to individuals from the modern or contemporary one. The possible sexual dimorphism variation in the morphology of cranial traits is not exempted from this concept, and this is the reason behind the need to evaluate it properly in various populations [7].

Overall, cranial morphological traits are sexually dimorphic and show different degrees of expression that vary within population groups. This is exploited for building ad hoc sex estimation methods that help in the construction of a biological profile in case of the recovery of unknown human remains. Since crania are overrepresented in forensic contexts [40,41], improving the accuracy of anthropological methods to apply to this anatomical region is crucial because sex is a foundational component of a biological profile and is often its starting point. However, both the general robustness and gracility of male and female skeletal remains, and the scale of sex-related differences, depend on the particular geographical population [42] and thus, methods developed from specific skeletal collections are limited in their applicability to underrepresented populations [43]. Therefore, the accuracy of current methods that predict biological profile characteristics should be adjusted according to the populations under study. This is not an impossible goal to accomplish given the numerous reference osteological collections created in the recent decades [44] on which to perform such research. One of the crucial issues for experts who deal with human remains in forensic contexts to consider and, overall, the key ‘push’ factor of our research into sexing methods—and of the present study on an Italian population—is to propose methods that are more accurate.

Our results on the sexual dimorphism expression of cranial traits in modern/contemporary Italians proved to be different from other collections/populations. The frequencies of the analyzed cranial traits, as well as the unsatisfactory accuracy of some of the discriminant functions created by Walker that were applied to our sample, proved this remarkable difference. In fact, all of the equations applied to our whole study sample highlighted the fact that the percentages of correct sex assessments were lower in general (74–85%) than the accuracy proved in the original population. The percentages of a correct assessment for female crania were the lowest (our study: 56–79%; Walker’s study: 78–86%), and, precisely, the predictive equation that combines the orbital margin and the mental eminence was the one performing worst (Table 4). On the contrary, very high accuracy was found for males: these logistic regression equations have resulted in even better performance for Italian males than the original male population on which Walker had developed them (our study: 90–96%; Walker’s study: 77–88%) [2]. This result is not a novelty in the literature: Oikonomopoulou et al. [25] found the same trend in a modern Greek population where all the Walker discriminant equations proved to have lower percentages of crania correctly classified in females (23–61%) and a reversed situation for male individuals (76–99%). The same trend was also found in modern Hispanics (correctly classified females using equation 1 by Walker: 70.4%; correctly classified males using equation 1 of Walker: 92.6%) [22] and White South Africans (correctly classified females: 31–62%; correctly classified males: 94–97 [21]. In contrast, diverse results were found in a Romanian population by Soficaru et al. [23], who also reported a high percentage of correct classifications for females (79–96%) in addition to males (86–97%). However, the latter tested the accuracy of Walker’s discriminant functions in a sample of individuals temporally closer to the White American/English original population (born in the 19th Century), stressing the importance of considering secular changes in addition to the geographical/ethnical ones when sex estimation methods are applied [4,18,45].

Intra- and inter-observer statistics proved the reliability of the scoring method, showing a good agreement between the analysts and the different trials (Cohen’s kappa values equal to or greater than 0.70). The highest agreement was observed for the glabella and the mastoid process in all tests (Cohen’s kappa values equal to or greater than 0.81), confirming the previous results from South African and American samples [18,21]. Such traits could be therefore considered characterized by the lowest subjectivity in the scoring and thus more reliable in sex diagnosis. Despite the general agreement observed, the intra-observer analysis revealed a slightly greater agreement between the trials carried out by the second analyst, who was characterized by a greater experience. This is a well-known finding in the literature. Lewis and Garvin [18] described similar results, suggesting the analyst experience as a factor influencing the assignment of the score and, therefore, the estimation of sex. However, the differences between the two analysts in our study were not so large, as attested by the high agreement observed.

Concerning the average scores for the five traits, our population showed significant differences between the scores assessed for males and females, proving a great sexual dimorphism, especially for the glabella and mastoid process, as is also deducible from our population-specific equation only including the two traits reported in Table 5. In general, in our study sample, the average scores observed for females (ranging from a minimum of 1.8 for glabella to a maximum of 2.3 for mental eminence and nuchal crest) were slightly higher than those reported by Soficaru et al. [23] (range 1.5 to 2.1) but slightly lower in comparison with those verified in White South African females, except for glabella, for which the average score was similar. On the contrary, the average scores noticed in Italian males were the highest (ranging from a minimum of 3.7 for the orbital margin to a maximum of 4.2 for glabella) when compared with those reported in the literature for the other populations: the values for White South African males were slightly lower (from a minimum of 3.4 for mental eminence to a maximum of 4.1 for the mastoid process), while they were considerably lower in Romanians (ranged from a minimum of 3.2 for orbital margin to a maximum of 3.7 for glabella and mental eminence). These results emphasize the presence of a great sexual dimorphism for cranial morphological traits between Italian males and females, and males included in our study’s sample present overall more robust traits (pronounced glabella, more robust mastoid process, and smoother orbital margin), explaining the reasons behind the large percentages of correct sex assessments for male Italian individuals when using the original discriminant formulae created by Walker.

Finally, we provided specific logistic regression equations for the Italian population. To be thorough, the equations for all possible combinations of cranial morphological traits were provided. However, the analyses revealed the best performing models with one or two cranial traits, showing no increasing accuracy with more combinations (more than two traits). In addition, the best performing models described included the traits associated with the highest agreement (i.e., the mastoid process and glabella), thus suggesting that these models are the most reliable in sex diagnosis. In general, by applying our newly-developed regression equations to the testing sample, the accuracy can reach higher satisfactory percentages, a result that makes these new models more suitable to be used in forensic contexts. This study does not represent the first-ever analysis using the same logistic regression approach for predicting sex in this skeletal collection: a previous study has focused on sexual dimorphism of craniometric variables [30] and provided population-specific equations based on skull metrical variables (linear and angular measurements) with satisfactory accuracies. However, the accuracy of these craniometric equations, 76% and 88%, respectively, for mandibular and cranial metric parameters, is lower than the accuracy achieved with the new morphological trait-based predictive models presented here. Furthermore, while most of the craniometric variables analyzed by the authors showed defined inter-population variability in terms of sexual dimorphism when compared with other populations, only a few cranial parameters were instead found to be constant independently from ancestry [30]. However, the weight of these constant variables proved different in the compared populations and thus needs to be verified in each specific population, highlighting once more the need to analyze the inter-population trend of sexual dimorphism for each variable considered, regardless of whether they are morphological or metric.

The present study has some limitations. In particular, the small number of young individuals, which included ages between 20 and 40 years and prevented the evaluation of the sexual dimorphism differences between different age classes. In fact, in the literature, age-related changes in the expression of cranial traits have been reported [2,9,46], suggesting a masculinization of some cranial traits with age [46]. Garvin and collaborators [9] reported a significant relationship between most cranial traits and age, even though only 13% of trait variation was explained by age. Moreover, older individuals were more prone to antemortem tooth loss and edentulous mandibles. However, no data are available so far on the changes of the site of interest (i.e., the mental eminence) in case of tooth loss [47], and further analyses are needed in the future that use samples that also include young individuals to clarify this statement. Another limitation is represented by the intrinsic subjectivity of the tested method, as it is a method based on morphological features. Although the agreement between the two analysts was high in this study, we cannot verify the subjectivity in scoring the traits between the analysts of the various studies here compared. This causes an important question to arise on which further investigations are needed: are the differences in the expression of certain traits found across diverse populations the result of sexual dimorphism variation or the subjectivity deriving from different anthropological training? That is surely a point to consider and to be verified in future. A further point to be considered as “food for thought” in the context of forensic anthropological investigation, especially in the current historical period characterized by human migration and heterogeneous countries in terms of population affinity, concerns the development of new models with very heterogeneous samples that are able to capture a significant range of human variation and are useful for predicting sex in contexts where no a priori knowledge is available [48].

Finally, the use of non-metric traits for sex determination is a basic and fast method in forensic anthropology and bioarchaeology. However, it is an old-fashioned approach that mostly relies on the analyst’s opinion and experience and, as such, it is a questionable method because it is more characterized by subjectivity. We wish that the application of modern techniques (3D models analysis, machine learning algorithms, or other alternative AI approaches, etc.) could be helpful for solving this task more objectively in the future.

## 5. Conclusions

The increasing number of known/identified skeletal collections is encouraging validation studies of sex estimation methods on various and diverse populations, as well as the development of additional predictive models. This is beneficial for many anthropological methods that allow for the profiling of human remains in forensics.

Since the sexual dimorphism of cranial morphological traits is population-specific, it is reasonable in forensic cases to employ the method proposed for the population from which the individual is supposed to come from. As in part suggested by the results of the present study and already reported in the literature, sexual dimorphism is affected by numerous factors, such as secular, ethnical, genetic, and environmental changes. Thus, when forensic anthropologists are dealing with real cases, they should always consider methods that are potentially validated on the proper population. The models here presented and built ad hoc for the Italian population allow for the prediction of the sex more accurately than the existing models and those based on craniometrics for the same population: they are weighted specifically for our sample, which might help avoid misclassifications in particular for female individuals.

Lastly, given the intrinsic subjectivity of the old-fashioned approaches as the one here proposed, when non-metric traits are considered in forensic cases, it is important not only to apply population-specific models when possible, but it is also preferable for more than one anthropologist to rate/score the traits.

## Figures and Tables

**Table 1:** Pearson’s chi-square test and corresponding p-value of the difference in distribution of each cranial morphological trait score between males and females according to whole, training, and validation samples.

Cranial Morphological Trait	Sample		
	Whole (Females = 84; Males = 93)	Training (Females = 60; Males = 64)	Validation (Females = 24; Males = 29)
	χ^2^ Test (*p*-Value)	χ^2^ Test (*p*-Value)	χ^2^ Test (*p*-Value)
Nuchal crest	85.1 (*p* < 0.01)	59.6 (*p* < 0.01)	26.0 (*p* < 0.01)
Mastoid process	84.6 (*p* < 0.01)	50.5 (*p* < 0.01)	36.5 (*p* < 0.01)
Orbital margin	90.9 (*p* < 0.01)	58.6 (*p* < 0.01)	32.7 (*p* < 0.01)
Glabella	115.5 (*p* < 0.01)	77.5 (*p* < 0.01)	39.1 (*p* < 0.01)
Mental eminence	76.7 (*p* < 0.01)	47.5 (*p* < 0.01)	31.8 (*p* < 0.01)

**Table 2:** Spearman’s correlation matrix ^a^ for morphological trait scores according to sex in the whole, training and validation samples.

Cranial Morphological Trait	Cranial Morphological Trait				
	Nuchal Crest	Mastoid Process	Orbital MARGIN	Glabella	Mental Eminence
**Whole sample**					
Nuchal crest	-	0.43 (*p* < 0.01)	0.36 (*p* < 0.01)	0.35 (*p* < 0.01)	<0.01 (*p* = 0.99)
Mastoid process	0.41 (*p* < 0.01)	-	0.41 (*p* < 0.01)	0.40 (*p* < 0.01)	0.36 (*p* < 0.01)
Orbital margin	0.39 (*p* < 0.01)	0.36 (*p* < 0.01)	-	0.44 (*p* < 0.01)	0.27 (*p* < 0.01)
Glabella	0.48 (*p* < 0.01)	0.38 (*p* < 0.01)	0.60 (*p* < 0.01)	-	0.39 (*p* < 0.01)
Mental eminence	0.50 (*p* < 0.01)	0.33 (*p* < 0.01)	0.38 (*p* < 0.01)	0.49 (*p* < 0.01)	-
**Training sample**					
Nuchal crest	-	0.47 (*p* < 0.01)	0.39 (*p* < 0.01)	0.44 (*p* < 0.01)	−0.03 (*p* = 0.81)
Mastoid process	0.49 (*p* < 0.01)	-	0.45 (*p* < 0.01)	0.37 (*p* < 0.01)	0.39 (*p* < 0.01)
Orbital margin	0.44 (*p* < 0.01)	0.41 (*p* < 0.01)	-	0.40 (*p* < 0.01)	0.28 (*p* = 0.02)
Glabella	0.55 (*p* < 0.01)	0.44 (*p* < 0.01)	0.69 (*p* < 0.01)	-	0.32 (*p* < 0.01)
Mental eminence	0.45 (*p* < 0.01)	0.40 (*p* < 0.01)	0.40 (*p* < 0.01)	0.47 (*p* < 0.01)	-
**Validation sample**					
Nuchal crest	-	0.33 (*p* = 0.08)	0.27 (*p* = 0.16)	0.13 (*p* = 0.52)	0.10 (*p* = 0.60)
Mastoid process	0.16 (*p* = 0.45)	-	0.32 (*p* = 0.09)	0.46 (*p* = 0.01)	0.27 (*p* = 0.11)
Orbital margin	0.25 (*p* = 0.23)	0.18 (*p* = 0.40)	-	0.59 (*p* < 0.01)	0.21 (*p* = 0.27)
Glabella	0.35 (*p* = 0.10)	0.20 (*p* = 0.34)	0.39 (*p* = 0.06)	-	0.56 (*p* < 0.01)
Mental eminence	0.62 (*p* < 0.01)	0.07 (*p* = 0.74)	0.33 (*p* = 0.12)	0.60 (*p* < 0.01)	-

^a^ The upper right side of matrices (in grey) refers to correlations for males, whereas the lower left side of matrices (in white) refers to correlations to females.

**Table 3:** Inter- and intra-observer agreement of cranial morphological trait scores.

	Cranial Morphological Trait				
	Nuchal Crest	Mastoid Process	Orbital Margin	Glabella	Mental Eminence
**Inter-observer agreement**					
Agreement (%)	55.4	60.7	58.3	65.5	61.7
Weighted Cohen’s kappa	0.74 (*p* < 0.01)	0.86 (*p* < 0.01)	0.75 (*p* < 0.01)	0.81 (*p* < 0.01)	0.73 (*p* < 0.01)
Agreement with a tolerance of ± 1 (%)	90.5	96.4	94.0	93.4	92.2
**Intra-observer agreement for analyst 1**					
Agreement (%)	55.4	60.7	50.0	67.9	67.9
Weighted Cohen’s kappa	0.70 (*p* < 0.01)	0.88 (*p* < 0.01)	0.76 (*p* < 0.01)	0.92 (*p* < 0.01)	0.81 (*p* < 0.01)
Agreement with a tolerance of ± 1 (%)	92.9	98.2	94.6	98.2	96.4
**Intra-observer agreement for analyst 2**					
Agreement (%)	59.6	68.1	78.7	83.0	68.1
Weighted Cohen’s kappa	0.85 (*p* < 0.01)	0.94 (*p* < 0.01)	0.90 (*p* < 0.01)	0.96 (*p* < 0.01)	0.87 (*p* = 0.01)
Agreement with a tolerance of ± 1 (%)	97.9	100	95.7	100	97.9

**Table 4:** Classification accuracy in predicting sex applying Walker’s equations on our study sample.

Combinations of Cranial Morphological Traits	Correctly Classified (%) Using Whole Sample			Sex Bias (%)
	Combined	Females	Males	
Nuchal crest, mastoid process	83.6	76.2	90.3	−14.1
Mastoid process, glabella	84.7	72.6	95.7	−23.1
Mastoid process, mental eminence	79.7	67.9	90.3	−22.4
Orbital margin, mental eminence	74.6	56.0	91.4	−29.2
Glabella, mental eminence	81.9	66.7	95.7	−29.0
Mastoid process, glabella, mental eminence	85.9	78.6	92.5	−13.9

**Table 5:** Discriminant analyses for predicting sex from all possible combinations of cranial morphological trait scores using logistic regression model.

Combinations of Cranial Morphological Traits	Estimates		AIC ^a^	Correctly Classified (%) Using Training Sample ^b^		Sex Bias (%)	Correctly Classified (%) Using Validation Sample ^b^		Sex Bias (%)
	Intercept	Cranial Morphological Traits Coefficient ^c^		Females	Males		Females	Males	
Nuchal crest	−5.95	**1.886**	105.5	90.0	70.3	19.7	87.5	75.9	11.6
Mastoid process	−3.442	**1.261**	118.7	75.0	84.4	−9.4	87.5	93.1	−5.6
Orbital margin	−4.349	**1.568**	111.0	75.0	90.6	−15.6	83.3	93.1	−9.8
Glabella	−4.764	**1.603**	89.5	75.0	92.2	−17.2	75.0	96.6	−21.6
Mental eminence	−3.662	**1.226**	108.7	60.0	85.9	−25.9	75.0	96.6	−21.6
Nuchal crest, mastoid process	−5.933	**1.369**, **0.586**	102.1	85.0	78.1	6.9	87.5	82.2	4.7
Nuchal crest, orbital margin	−6.728	**1.296**, **0.935**	100.0	86.7	82.8	3.9	87.5	86.2	1.3
Nuchal crest, glabella	−6.437	**0.916**, **1.192**	107.6	90.0	89.1	0.9	91.7	93.1	−1.4
Nuchal crest, mental eminence	−7.023	**1.528**, **0.702**	85.1	85.0	85.9	−0.9	87.5	86.2	1.3
Mastoid process, orbital margin	−5.006	**0.716**, **1.092**	103.9	85.0	87.5	−2.5	87.5	96.6	−9.1
Mastoid process, glabella	−5.558	**0.575**, **1.335**	86.5	90.0	90.6	−0.6	87.5	96.6	−9.1
Mastoid process, mental eminence	−4.571	**0.932**, **0.672**	112.2	80.0	81.2	−1.2	91.7	93.1	−1.4
Orbital margin, glabella	−5.491	0.59 ^d^, **1.272**	88.5	83.3	90.6	−7.3	87.5	96.6	−9.1
Orbital margin, mental eminence	−5.325	**1.223**, **0.641**	105.5	83.3	87.5	−4.2	87.5	89.7	−2.2
Glabella, mental eminence	−5.65	**1.43**, 0.466 ^d^	88.1	83.3	90.6	−7.3	83.3	96.6	−13.3
Nuchal crest, mastoid process, orbital margin	−6.566	**1.099**, 0.287, **0.818**	97.0	86.7	89.1	−2.4	91.7	89.7	2.0
Nuchal crest, mastoid process, glabella	−6.489	0.682, 0.342, **1.13**	85.8	90.0	89.1	0.9	91.7	93.1	−1.4
Nuchal crest, mastoid process, mental eminence	−6.851	**1.303**, 0.324, **0.591**	98.4	81.7	89.1	−7.4	87.5	89.7	−2.2
Nuchal crest, orbital margin, glabella	−6.757	**0.809**, 0.401, **1.014**	85.9	90.0	90.6	−0.6	91.7	93.1	−1.4
Nuchal crest, orbital margin, mental eminence	−7.226	**1.171**, **0.721**, 0.478 ^d^	94.3	85.0	89.1	−4.1	87.5	86.2	1.3
Nuchal crest, glabella, mental eminence	−6.891	**0.812**, **1.084**, 0.346	85.4	88.3	92.2	−3.9	91.7	93.1	−1.4
Mastoid process, orbital margin, glabella	−5.822	0.477 ^d^, 0.343, **1.183**	87.7	93.3	90.6	2.7	87.5	96.6	−9.1
Mastoid process, orbital margin, mental eminence	−5.468	**0.551**, **0.956**, 0.428	103.2	86.7	87.5	−0.8	91.7	93.1	−1.4
Mastoid process, glabella, mental eminence	−5.886	0.459, **1.278**, 0.271	87.6	90.0	89.1	0.9	87.5	96.6	−9.1
Orbital margin, glabella, mental eminence	−5.987	0.444, **1.206**, 0.372	88.6	88.3	89.1	−0.8	87.5	96.6	−9.1
Nuchal crest, mastoid process, orbital margin, glabella	−6.671	0.658, 0.258, 0.290, **1.016**	87.2	90.0	90.6	−0.6	91.7	93.1	−1.4
Nuchal crest, mastoid process, orbital margin, mental eminence	−7.128	**1.106**, 0.115, **0.683**, 0.447 ^d^	96.2	85.0	89.1	−4.1	87.5	86.2	1.3
Nuchal crest, mastoid process, glabella, mental eminence	−6.801	0.68, 0.229, **1.064**, 0.269	86.9	90.0	92.2	−2.2	91.7	93.1	−1.4
Nuchal crest, orbital margin, glabella, mental eminence	−7.043	0.749 ^d^, 0.307, **0.963**, 0.293	86.7	88.3	90.6	−2.3	91.7	93.1	−1.4
Mastoid process, orbital margin, glabella, mental eminence	−6.064	0.389, 0.291, **1.155**, 0.238	89.0	90.0	89.1	0.9	87.5	96.6	−9.1
Nuchal crest, mastoid process, orbital margin, glabella, mental eminence	−6.94	0.665, 0.162, 0.248, **0.972**, 0.248	88.5	88.3	92.2	−3.2	91.7	93.1	−1.4

^a^ AIC, Aikake’s information criterion. ^b^ Predicted probabilities greater than 0.5 are more likely to be males, while predicted probabilities lower than 0.5 are more likely to be females; ^c^ Estimates with a significance level of 0.05 are reported in bold; ^d^ Estimates with a significance level of 0.10.

## Data Availability

The data presented in this study are available on request from the corresponding author.
